# *Ganoderma lucidum* spore powder alleviates rheumatoid arthritis-associated pain hypersensitivity through inhibiting accumulation, N1 polarization, and ROS production of neutrophils in mice

**DOI:** 10.3389/fimmu.2025.1569295

**Published:** 2025-04-30

**Authors:** Sen Huang, Zhaochun Zhan, Fei Xu, Xiaolin Liu, Zhenning Fang, Wenbo Wu, Zhile Liang, Guoguo Liu, Mengyuan Wang, Helena Soares da Silva, Xin Luo, Kai Mo

**Affiliations:** ^1^ Department of Anesthesiology, Zhujiang Hospital, Southern Medical University, Guangzhou, China; ^2^ Key Laboratory of Mental Health of the Ministry of Education, Guangdong-Hong Kong-Macao Greater Bay Area Center for Brain Science and Brain-Inspired Intelligence, Guangdong-Hong Kong Joint Laboratory for Psychiatric Disorders, Guangdong Province Key Laboratory of Psychiatric Disorders, Guangdong Basic Research Center of Excellence for Integrated Traditional and Western Medicine for Qingzhi Diseases, Department of Neurobiology, School of Basic Medical Sciences, Southern Medical University, Guangzhou, China; ^3^ Department of Anesthesiology, Shunde Hospital, Southern Medical University, Foshan, China; ^4^ Institute of Perioperative Medicine and Organ Protection, Zhujiang Hospital, Southern Medical University, Guangzhou, China

**Keywords:** rheumatoid arthritis, pain, neutrophils, *Ganoderma lucidum* spore powder, reactive oxygen species

## Abstract

**Introduction:**

Rheumatoid arthritis (RA) is a chronic condition characterized by joint pain that significantly impairs patients’ work and daily lives. The limited understanding of the pathological mechanisms underlying RA-related pain poses challenges for effective clinical pain management. *Ganoderma lucidum* spore powder (GLSP) has demonstrated therapeutic benefits in various diseases, with no reported toxicity or adverse effects.

**Methods:**

This study investigates the role of neutrophils in the pathological mechanisms of RA-related pain using collagen-induced arthritis (CIA) mice and an *ex vivo* neutrophil model. A combination of techniques, including animal models, flow cytometry, behavioral testing, cell adoptive transfer, and network pharmacology analysis, was employed to evaluate the effects and targets of GLSP on pain symptoms and neutrophil activity in CIA mice.

**Results:**

Flow cytometric analysis revealed the accumulation and activation of neutrophils in the paws of CIA mice. Furthermore, the levels of pro-inflammatory CD95^+^ neutrophil subpopulations (N1 state) and ROS^+^ cells in the affected paws were positively correlated with the severity of mechanical allodynia and heat hyperalgesia observed in these mice. Our findings indicate that oral administration of GLSP significantly alleviates joint destruction, paw swelling, and pain hypersensitivity in CIA mice. Notably, GLSP reversed CIA-induced neutrophil accumulation, N1 polarization, and reactive oxygen species (ROS) production. Both network pharmacology target prediction and *in vivo/in vitro* experimental validation indicated that GLSP inhibits N1 polarization and ROS production in neutrophils by modulating the TNF-α signaling pathway, thus exerting RA-specific analgesic effects.

**Discussion:**

In summary, this study offers new insights into the pathological mechanisms of RA-related pain and demonstrates that neutrophil accumulation, N1 polarization, and ROS production contribute to RA-related pain. GLSP alleviates RA-related pain by inhibiting the pro-inflammatory phenotype of neutrophils, highlighting its potential for clinical translation in the treatment of RA.

## 1 Introduction

Rheumatoid arthritis (RA) is a prevalent autoimmune disease characterized by erosive joint inflammation, leading to cartilage and bone destruction, joint deformity, functional disability, and extra-articular manifestations. Despite its prevalence, the pathogenesis of RA remains incompletely elucidated (1, 2). Epidemiological data indicate that, globally, the age-standardized point prevalence and annual incidence rates of RA were 246.6 (95% UI 222.4 to 270.8) and 14.9(95% UI 13.3 to 16.4), respectively, in 2017 (3). Disability rates rise significantly with disease progression, contributing to RA’s designation as a severely debilitating condition (4). Symmetrical polyarticular pain, a hallmark symptom of RA, impairs both physical function and social participation, making pain relief a primary therapeutic goal (1, 2). Early and effective pain management facilitates functional exercise, prevents late-stage joint stiffness and deformity, and is therefore essential in RA management (5, 6). Disease-modifying antirheumatic drugs (DMARDs), encompassing conventional synthetic and biologic agents, are the cornerstone of RA treatment. While DMARDs can retard disease progression, achieving complete remission remains elusive for many patients due to various factors (7). For example, first-line immunosuppressants such as methotrexate, methylprednisolone, and cyclosporin A offer limited analgesia and may induce adverse effects with long-term use (8). During active disease, nonsteroidal anti-inflammatory drugs (NSAIDs) provide short-term pain relief, but long-term efficacy is limited by a ceiling effect and potential adverse events. In periods of low inflammatory activity, NSAID efficacy diminishes, and approximately 40% of patients require opioid analgesics (9). Biologics demonstrate superior efficacy compared to conventional DMARDs, but their high cost restricts accessibility. Overall, the incomplete understanding of RA-related pain mechanisms hinders the development of targeted analgesic therapies.

In RA, the recruitment of numerous signaling molecules and the infiltration of innate and adaptive immune cells into the synovium of affected joints trigger functional alterations that contribute to disease pathogenesis (10, 11). Previous research from our group has highlighted the role of neuro-immune interactions in chronic pain mechanisms (12–14). In the context of RA, synovial immune cells may release pain mediators that activate peripheral sensory neurons, initiating the pain cascade. Therefore, investigating the contribution of these immune cells to RA-related pain may further elucidate disease mechanisms. Neutrophils, the predominant myeloid leukocytes and primary effectors of innate immunity in various disease states (15), undergo functional phenotypic changes, termed “polarization,” through interactions with signaling molecules within the affected tissue microenvironment. Neutrophils exhibit two primary functional states: the pro-inflammatory N1 phenotype (marked by CD95) and the anti-inflammatory N2 phenotype (marked by CD206) (16). Studies indicate that neutrophils contribute to RA pathogenesis through the release of cytokines and chemokines, promotion of protein citrullination, release of neutrophil extracellular traps (NETs), and production of reactive oxygen species (ROS) within affected joints (17, 18). However, the precise role of neutrophils in RA-related pain remains to be fully defined. Our research has demonstrated a positive correlation between neutrophil accumulation, N1 polarization within the synovium of RA patients, and pain scores. Furthermore, neutrophils in the joints of mice with collagen-induced arthritis (CIA) exhibit a similar phenotype to that observed in human RA. These findings suggest that synovial neutrophils may represent a novel cellular mechanism contributing to RA-related pain and a potential therapeutic target.

Nutraceuticals and functional foods contain numerous bioactive compounds that, beyond their nutritional value, may offer therapeutic or preventative benefits in various diseases, such as hypertension (19). *Ganoderma lucidum*, a traditional Chinese medicine, is recognized for its antioxidant, anti-inflammatory, and antitumor properties (20, 21). The absence of reported toxicity or adverse effects associated with *Ganoderma lucidum*-derived compounds has fueled its increasing popularity as a dietary supplement, natural therapy, and health-enhancing food, attracting attention from both industry and scientific communities (22). *Ganoderma lucidum* spore powder (GLSP), due to its ease of consumption and the extractability of bioactive components via water or ethanol soaking after removal of the spore coat, has become a focus of research. Numerous studies have demonstrated that water or ethanol extracts of GLSP exhibit antitumor, anti-inflammatory, anti-dementia, and anti-arteriosclerotic effects (23–26). The present study investigates the analgesic effects of GLSP on RA-related pain using *in vivo* and *in vitro* models. Our results demonstrate that oral GLSP administration significantly ameliorates pain hypersensitivity in CIA mice and reverses neutrophil N1 polarization and ROS production. These findings provide new insights into the pathophysiology of RA-related pain and offer potential therapeutic strategies for its management.

## 2 Materials and methods

### 2.1 Ethics statement

Male C57BL/6 mice (aged 8–16 weeks) were purchased from Guangdong Zhiyuan Biomedical Technology Co., Ltd. (Guangzhou, China) and Zhaoqing Ruisi Yuan Biotech Co., Ltd. (Zhaoqing, China). The mice were maintained in a controlled environment (12-hour light/dark cycle, 44–65% humidity), with ad libitum access to a standard rodent diet and water. All animal procedures adhered to institutional and national guidelines for animal care and use, with measures implemented to minimize the duration of experiments and alleviate animal suffering. Mice reaching the experimental endpoint were euthanized either by exsanguination under deep anesthesia with 3% isoflurane or by CO_2_ inhalation at a flow rate of 50% of the chamber volume per minute. Euthanasia was conducted humanely to ensure minimal pain, fear, and stress. All protocols were approved by the Animal Ethics Committee of Southern Medical University (Approval No. SMUL202403011).

### 2.2 Induction of collagen-induced arthritis

Collagen-induced arthritis (CIA) was induced in C57BL/6 mice following established protocols (27). Briefly, native chicken type II collagen (Chondrex) was emulsified with an equal volume of complete Freund’s adjuvant (Chondrex) in ice. Following anesthesia with 3% isoflurane, each mouse received a single 100 μl intradermal injection at the tail base, containing 200 μg of collagen. Arthritis severity was assessed using a four-point scale: 0, normal; 1, mild swelling and/or erythema of the digits; 2, moderate swelling and erythema extending from the ankle to the tarsus; 3, marked swelling from the ankle to the metatarsophalangeal joints; and 4, maximal swelling and erythema involving the ankle, foot, and digits, with resultant deformity and/or ankylosis. The total clinical score (0-16) was calculated by summing the scores for each limb. All assessments were performed in a blinded manner.

### 2.3 Behavioral tests

All behavioral tests were performed blinded, using established methods that permitted animals to escape noxious stimuli. For von Frey testing, mice were habituated for at least 48 hours prior to testing in elevated cages with wire mesh floors, under controlled temperature and humidity. Von Frey filaments (0.02–2.56 g, Stoelting) of logarithmically increasing stiffness were applied to the plantar surface of the hind paw, and paw withdrawal thresholds (PWTs) were determined using the up-down method (28). Thermal hyperalgesia was assessed using the Hargreaves test (Hargreaves apparatus, IITC Life Science), with a 20-second cutoff to prevent tissue damage. Cold allodynia was assessed by applying 20 μl of acetone to the plantar hind paw, and responses were scored as follows: 0, no response; 1, brief withdrawal, stamping, or flicking; 2, prolonged withdrawal or repeated flicking; and 3, repeated flicking and licking (29). For thermal preference testing, mice were placed on a metal plate with a continuous temperature gradient (5–56°C) and their movement was video-recorded (Bioseb) for 90 minutes. Following a 30-minute acclimation period, time spent in each temperature zone over the subsequent 60 minutes was recorded. Two mice were tested concurrently in separate channels (30).

Paw thickness was measured with calipers before model induction to establish baseline values. Measurements were repeated on days 21 and 10 post-immunization and post-treatment, respectively. Paw swelling was calculated as the percentage change from baseline.

### 2.4 Reagents

A list of reagents for this study is showed in **Supplementary Table S1**.

### 2.5 Drug administration in mice

GLSP was formulated as a suspension at a concentration of 50 mg/ml in distilled water for oral gavage administration in mice. In the CIA mouse model, oral gavage with drug treatment began at 3 weeks post-immunization and continued every other day until the end of the experiment. Control and sham groups received an equivalent volume of double-distilled water by oral gavage. For intraplantar (i.pl.) injections, mice were briefly anesthetized with 3% isoflurane, and 10 μl of drug or 1 × 10⁵ cells in 10 μl PBS were administered via a 29G needle into the plantar surface of the hind paw.

### 2.6 Cell culture and treatments

HL-60 cells (BeNa Culture Collection, Henan, China) were differentiated into neutrophil-like dHL-60 cells using 1.3% DMSO. dHL-60 cells were cultured in DMEM supplemented with 10% FBS, streptomycin (100 mg/mL), and penicillin (100 U/mL) at 37°C in a humidified 5% CO2 atmosphere. To mimic the inflammatory environment of rheumatoid arthritis, dHL-60 cells were stimulated with 40 ng/mL recombinant human TNFα for 6 hours.

### 2.7 Extraction of active components from GLSP and chemical analysis

GLSP was soaked in 95% ethanol (50 mg/ml) or distilled (50 mg/ml) water for 24 hours, followed by ultrasonic extraction repeated three times for 40 minutes each. The resulting extract was filtered through a cell strainer to remove the residue, and the extract was concentrated to prepare the samples. The components of GLSP extract were identified using HPLC-QTOF-MS/MS.

### 2.8 Prediction of drug targets for GLSP extract

The SMILES notation of GLSP was obtained using PubChem (https://pubchem.ncbi.nlm.nih.gov/). Subsequently, this SMILES notation was uploaded to the Swiss Target Prediction database (http://www.swisstargetprediction.ch/), the Super-PRED database (https://prediction.charite.de/index.php) and the targetnet (http://targetnet.scbdd.com/calcnet/index/) to identify the targets of GLSP.

### 2.9 Prediction of targets for RA

RA-related targets were identified from several databases, including the GeneCards database (https://www.genecards.org/), DisGeNET database (https://www.disgenet.org/home/).

### 2.10 Construction of the protein-protein interaction network, gene ontology and Kyoto encyclopedia of genes and genomes enrichment analyses

PPI network analysis of the intersection targets between GLSP and RA was performed using the STRING database (https://string-db.org/). A minimum interaction score of >0.7 (scale: 0-1) was applied. Hub genes within the network were identified, and the network was visualized using Cytoscape 3.9.0. Key targets of GLSP for the treatment of RA were identified through CentiScaPe analysis utilizing Degree, Closeness, and Betweenness algorithms. GO and KEGG enrichment analyses were conducted using the ClusterProfiler package in R (version 4.4).

### 2.11 Molecular docking

The crystal structure of PTGS2 (PDB ID: 3HS5), MMP9 (PDB ID: 1L6J), XIAP (PDB ID: 8W59) and MMP3 (PDB ID: 1G49) was obtained from the Protein Data Bank. The energy of GLSP extract was minimized to a local minimum using the MMF94X force field. The AutoDock Tools version 1.5.7 package was employed to generate the docking input files and analyze the docking results, which were then visualized using PyMOL software.

### 2.12 Western blotting

To evaluate the protein levels of CD95 and CD206, dHL-60 (1×10⁶ cells) were washed with cold PBS after stimulation and lysed using RIPA buffer (1 mM PMSF). The cell lysates were centrifuged at 12,000 × g for 10 minutes at 4°C, separated by SDS-PAGE, and subsequently transferred to PVDF membranes. Rabbit polyclonal CD95 antibody (Proteintech, 1:1000 dilution), rabbit polyclonal CD206 antibody (Proteintech, 1:1000 dilution), and mouse polyclonal β-actin antibody (Proteintech, 1:1000 dilution) were used as primary antibodies. Goat anti-rabbit IgG HRP (Proteintech, 1:4000 dilution) and goat anti-mouse IgG HRP (Proteintech, 1:4000 dilution) served as secondary antibodies. Protein levels were semi-quantified using ImageJ and normalized to the corresponding controls.

### 2.13 Immunohistochemical assay

Following deep isoflurane anesthesia, mice were transcardially perfused with PBS, followed by 4% paraformaldehyde.Paws and L3-L5 dorsal root ganglia (DRG) were harvested and post-fixed. DRG were dehydrated in a 20–30% sucrose gradient, embedded in OCT (Tissue-Tek), and cryosectioned at 14 μm. Sections were immunostained for Trpv1 (Alomone Labs) and CGRP (Immunostar), followed by incubation with fluorescent secondary antibodies. DRG neurons were visualized by Nissl staining (Molecular Probes) using a Nikon A1R/A1+ confocal microscope (NIS-Elements AR).

Paws were paraffin-embedded, sectioned at 10 μm, and stained with hematoxylin and eosin (H&E) to assess histological changes and fibrosis. A blinded scoring system (0-9, with a possible +1) was used to evaluate the following (1): synovial hyperplasia and cartilage inflammation (1 point - mild; 2 points - severe) (2); pannus formation (3 points - mild; 4 points - severe); (3) periarticular space loss, bone-connective tissue adhesion, and partial bone destruction (5 points - mild; 6 points - severe); (4) joint space loss and bone destruction/fusion (7 points - mild; 8 points - severe); (5) ankle fibrosis (9 points); (6) bone marrow inflammation (+1 point).

### 2.14 Flow cytometry

Following anesthesia, paws were harvested, minced, and digested with collagenase IV in RPMI 1640 medium at 37°C for 1.5 h. The digested tissue was passed through a 70 μm cell strainer. Blood was collected in heparinized tubes, and red blood cells were lysed. Single-cell suspensions were prepared from paws and blood and stained for flow cytometry. Live CD45^+^ leukocytes were gated based on forward and side scatter (FSC/SSC) and doublet exclusion. The following cell populations were quantified among these leukocytes: macrophages (CD11b^+^F4/80^+^), T cells (CD3^+^), B cells (CD19^+^), dendritic cells (CD11b^+^CD11c^+^), and neutrophils (CD11b^+^Ly6G^+^). Neutrophil polarization status was assessed by identifying N1 (CD11b^+^Ly6G^+^CD95^+^) and N2 (CD11b^+^Ly6G^+^CD206^+^) neutrophils. For *in vitro* experiments, cells were harvested, washed with PBS, and prepared into single-cell suspensions. Neutrophils were stained with antibodies against CD45, CD11b, and CD66b. N1 and N2 neutrophils were identified using antibodies against CD95 and CD206, respectively. All antibodies were purchased from BioLegend (San Diego, CA, USA). Intracellular reactive oxygen species (ROS) levels were measured by incubating single-cell suspensions with H_2_DCFDA (10 μM) for 15 min. All flow cytometry data were acquired on a BD LSRFortessa X-20 (BD Biosciences) and analyzed using FlowJo software.

### 2.15 Statistical analysis

Data are presented as mean ± SEM. Statistical analyses were performed using GraphPad Prism 9. Data normality was assessed using the Kolmogorov-Smirnov test. One-way, two-way, or repeated measures (RM) ANOVA followed by Tukey’s *post hoc* test, or unpaired/paired two-tailed Student’s t-test was used for normally distributed data. The nonparametric Mann-Whitney U test was used for non-normally distributed data. p < 0.05 was considered statistically significant. Pearson correlation analysis was performed after confirming normality. Detailed statistics are available upon request.

## 3 Result

### 3.1 *Ganoderma lucidum* spore powders alleviate RA-associated joint swelling and pain

**Figure 1:**
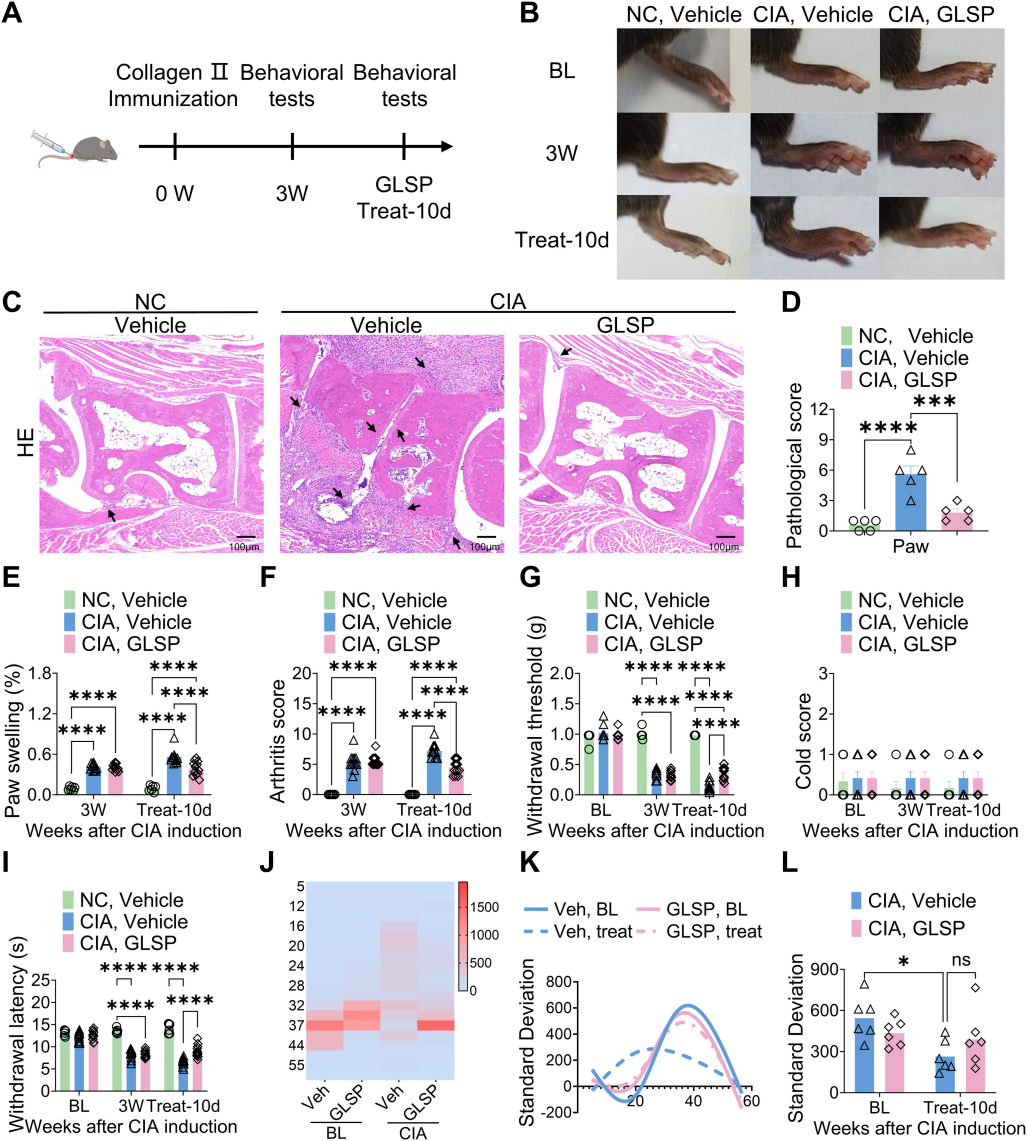
*Ganoderma lucidum* spore powder demonstrates the potential to alleviate joint damage and pain associated with RA. **(A)** Schematic representation of the experimental protocol. **(B)** Representative images illustrating the paws of mice from distinct experimental groups. **(C, D)** Hematoxylin and eosin (HE) staining data indicate that GLSP mitigates joint damage in CIA mice, with damage highlighted by arrows. The scale bar measures 100 µm. **(E, F)** GLSP treatment reduces paw swelling and arthritis scores induced by CIA. **(G–I)** GLSP treatment alleviates mechanical allodynia and thermal hyperalgesia in CIA mice, but has no effect on cold allodynia. **(J–L)** GLSP treatment reverses CIA-induced changes in temperature preference in mice. Data are mean ± SEM. *p < 0.05, **p < 0.01, ***p < 0.001, and ****p < 0.0001, one-way ANOVA assay followed by Tukey’s *post hoc* test **(D)**, two-way ANOVA assay followed by Tukey’s *post hoc* test **(E–I, L)**. NS, no significance.

*Ganoderma lucidum* spore powder (GLSP), derived from *Ganoderma lucidum*, has demonstrated significant anti-inflammatory and antioxidant effects, and therapeutic benefits in various diseases (23, 25, 31–33). However, its protective effects and mechanisms against RA remain to be elucidated. This study investigated the therapeutic potential of GLSP on RA-associated inflammation and pain. Beginning three weeks after immunization, naive mice and mice with collagen-induced arthritis (CIA) received oral GLSP (100 mg/kg/day) for 10 days (**Figure 1A**). Three weeks post-immunization, CIA mice exhibited significant paw swelling and elevated arthritis index scores. Following 10 days of treatment, the vehicle group showed further increases in paw swelling and arthritis index scores, whereas GLSP treatment significantly reduced both (**Figures 1B, E, F**). Histological analysis (hematoxylin-eosin staining) revealed that GLSP alleviated the characteristic RA pathological changes in the hind paws of CIA mice, including bone destruction and synovial hyperplasia (**Figures 1C, D**). Behavioral tests showed that 3 weeks post-immunization, CIA mice exhibited significant mechanical allodynia and thermal hyperalgesia, but not cold allodynia, compared with naive mice (**Figures 1G–I**). After 10 days of treatment, mechanical allodynia and thermal hyperalgesia were exacerbated in vehicle-treated CIA mice, while GLSP treatment significantly alleviated these pain symptoms (**Figures 1G–I**). Thermal preference was assessed using a thermal gradient test (30). Naive mice preferred the warmer temperature zone (34–43°C), whereas CIA mice exhibited a preference for the colder zone (16–32°C). GLSP treatment restored the thermal preference of CIA mice to the warmer zone (34–43°C) (**Figures 1J, K**), likely reflecting the alleviation of thermal hyperalgesia, consistent with the lack of effect on cold allodynia. Furthermore, assessment of dwell time in each temperature zone revealed that CIA mice spent less time in each zone, indicative of increased discomfort (**Figure 1L**). GLSP treatment significantly reduced this discomfort-related behavior (**Figure 1L**). Together, our findings implicate that GLSP may exert beneficial action on RA-associated inflammation and pain symptoms.

### 3.2 GLSP treatment alleviates the hyperexcitability of DRGs neurons in CIA mice

**Figure 2:**
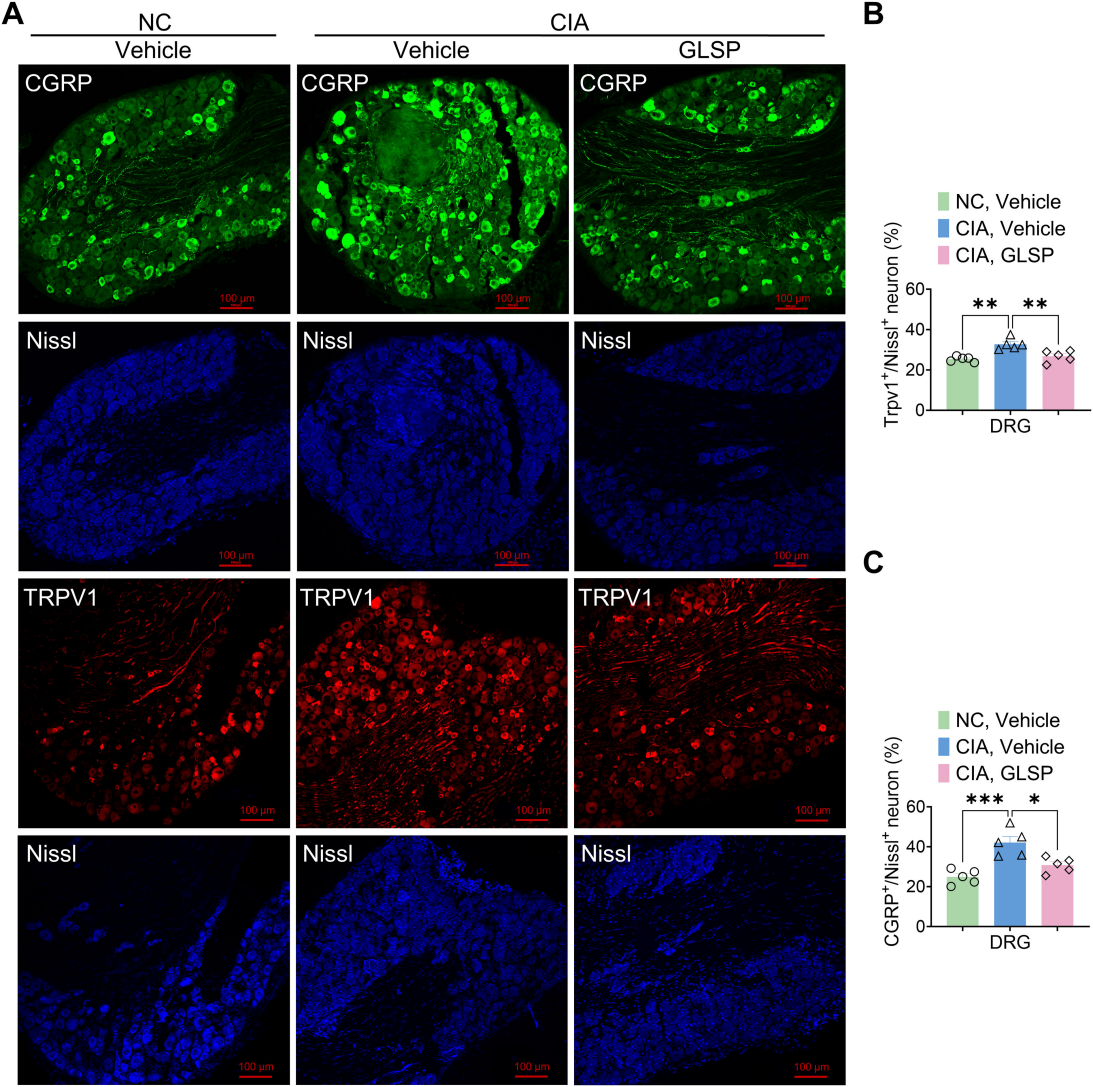
GLSP treatment alleviates the hyperexcitability of DRGs neurons in CIA mice. **(A)** Representative images of immunofluorescence (IF) staining for lumbar DRGs neurons from various experimental groups of mice are presented. **(B)** IF data reveals that GLSP attenuates the CIA-induced upregulation of TRPV1 expression in DRGs neurons. **(C)** IF data indicate that GLSP decreases the secretion of CGRP elevated by CIA in DRGs neurons. Data are mean ± SEM. *p < 0.05, **p < 0.01, ***p < 0.001, and ****p < 0.0001, one-way ANOVA assay followed by Tukey’s *post hoc* test **(B, C)**.

To investigate the neuronal mechanisms underlying the analgesic effects of GLSP in RA, we first evaluated its impact on sensory neurons in the dorsal root ganglion (DRG). Transient receptor potential vanilloid 1 (TRPV1) is a non-selective cation channel predominantly expressed in nociceptors, which detects noxious stimuli and facilitates calcium influx, thereby transducing pain signals (34). Calcitonin gene-related peptide (CGRP), a neuropeptide inducibly expressed by peptidergic nociceptors, modulates immune responses and neuronal activity (35). Numerous studies have indicated that TRPV1 and CGRP contribute to the pathology of RA-associated pain (36, 37). In this study, immunofluorescence analysis revealed that, compared with naive mice, collagen type II immunization significantly increased TRPV1 and CGRP expression in DRG neurons of vehicle-treated CIA mice at 4 weeks, suggesting that RA induces nociceptor sensitization (**Figures 2A–C**). Notably, GLSP treatment reversed this upregulation, indicating its potential to alleviate neuronal hyperexcitability in the DRG of CIA mice (**Figures 2A–C**). In summary, GLSP mitigates mechanical allodynia and thermal hyperalgesia by attenuating the sensitization of sensory neurons in the DRG of CIA mice.

### 3.3 GLSP exerts its therapeutic effects by reducing neutrophil accumulation and the polarization of the N1 pro-inflammatory phenotype in the paws of CIA mice

**Figure 3:**
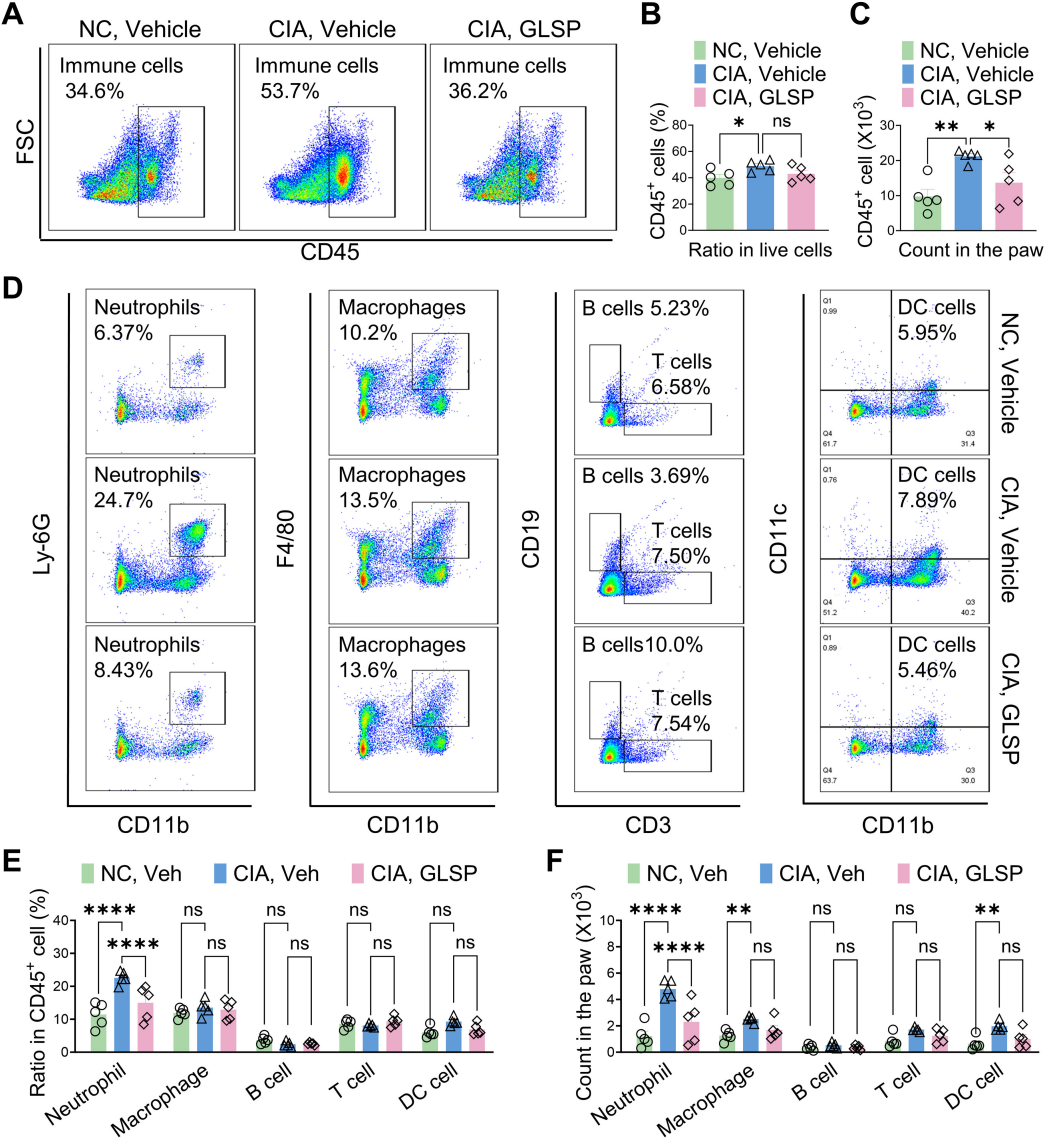
GLSP reduced neutrophil accumulation in the paws of CIA mice. **(A)** Representative flow cytometry images of affected paw samples from CIA and naïve mice. **(B, C)** GLSP decreases the proportion and number of CD45^+^ leukocytes in the affected paw tissues of CIA mice. **(D)** Representative flow cytometry images depict various immune cell populations within paw samples from CIA mice and naive mice. **(E, F)** GLSP reduced the proportion and count of neutrophils, while not affecting other immune cell populations in the affected paws of CIA mice. Data are mean ± SEM. *p < 0.05, **p < 0.01, ***p < 0.001, and ****p < 0.0001, one-way ANOVA assay followed by Tukey’s *post hoc* test **(B, C)**, two-way ANOVA assay followed by Tukey’s *post hoc* test **(E, F)**. NS, no significance.

Rheumatoid arthritis (RA) is an autoimmune disease characterized by immune cell dysfunction and the accumulation of various immune cells, including neutrophils, macrophages, T cells, B cells, and dendritic cells, within the synovium, leading to joint destruction (38). To investigate the immunomodulatory effects of GLSP, we analyzed immune cell populations in the paws of collagen-induced arthritis (CIA) mice using flow cytometry (**Supplementary Figure S1A**). We identified specific immune cell populations using antibodies against neutrophils (CD45^+^CD11b^+^Ly6G^+^), macrophages (CD45^+^CD11b^+^F4/80^+^), T cells (CD45^+^CD3^+^), B cells (CD45^+^CD19^+^), and dendritic cells (CD45^+^CD11b^+^CD11C^+^) (27, 39) (**Supplementary Figure S1B**). Compared to the control group, GLSP treatment significantly reduced the total number of CD45^+^ leukocytes in the hind paws of CIA mice without affecting the proportion of these leukocytes (**Figures 3A–C**). This reduction was primarily driven by a decrease in Ly-6G^+^ neutrophils, with no significant changes observed in other immune cell populations (**Figures 3D–F**). These findings suggest that GLSP may exert its therapeutic effects on RA by targeting synovial neutrophils.

**Figure 4:**
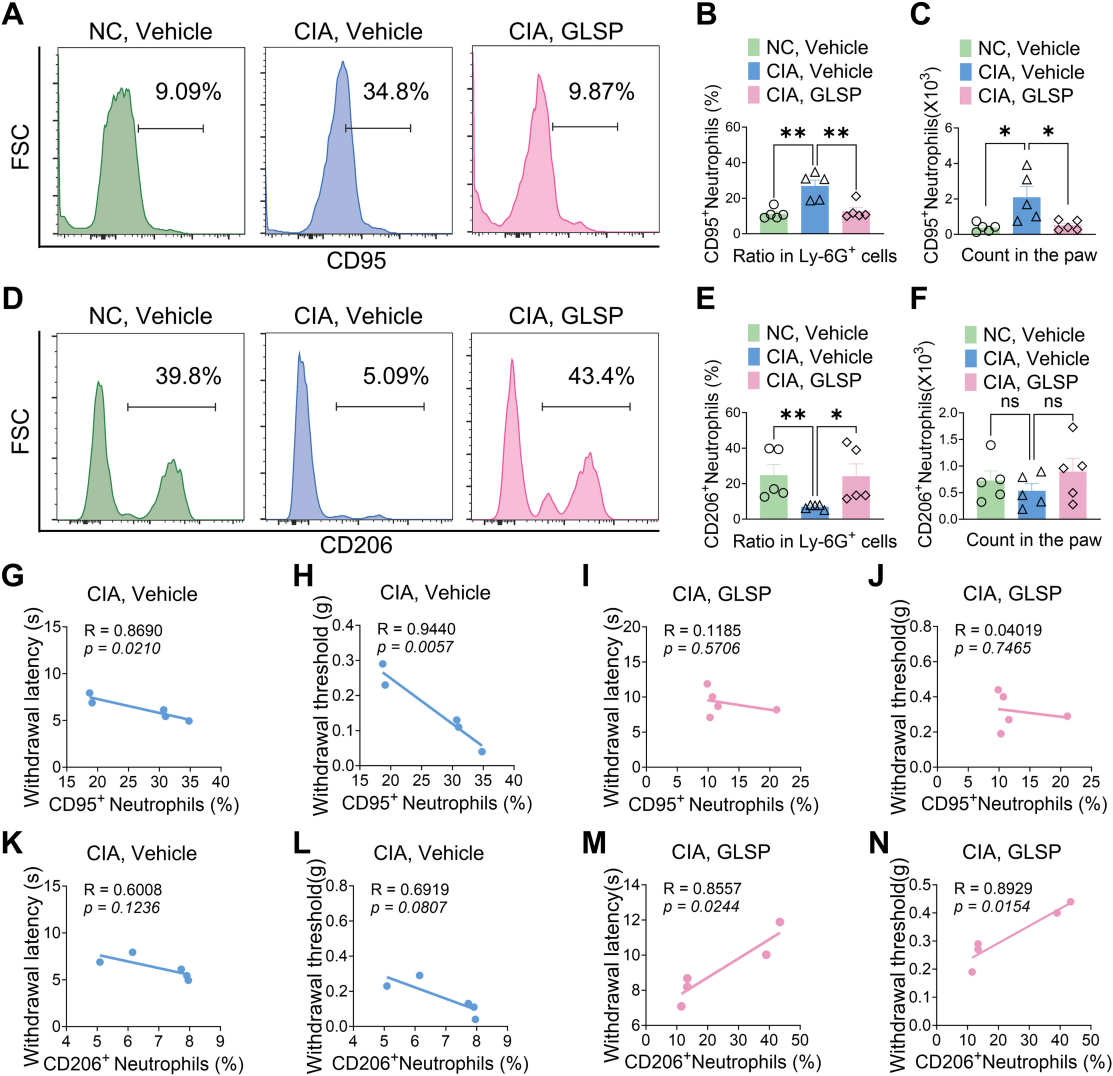
GLSP treatment reversed the N1-polarized state of neutrophils in the affected paws of CIA mice. **(A–C)** GLSP treatment reduced both the proportion and absolute count of N1-polarized (CD95^+^) neutrophils in the paws of mice with CIA. **(D–F)** GLSP treatment upregulated both the proportion and number of N2-state (CD206^+^) neutrophils within the paws of CIA mice. **(G, H)** In vehicle-treated CIA mice, N1 polarization of joint neutrophils exhibits a negative correlation with mechanical and thermal pain thresholds. **(I, J)** In GLSP-treated CIA mice, N1 polarization of joint neutrophils demonstrates no correlation with mechanical or thermal pain thresholds. **(K, L)** In vehicle-treated CIA mice, N2 polarization of joint neutrophils shows no significant correlation with mechanical or thermal pain thresholds. **(M, N)** N2 polarization of joint neutrophils in GLSP-treated CIA mice correlates positively with both mechanical and thermal pain thresholds. Data are mean ± SEM. *p < 0.05, **p < 0.01, ***p < 0.001, and ****p < 0.0001, one-way ANOVA assay followed by Tukey’s *post hoc* test **(B, C, E, F)**, Pearson correlation analysis **(G–N)**. NS, no significance.

Neutrophils are key players in the early inflammatory response in RA, rapidly infiltrating the synovium and contributing to joint inflammation (1). These cells can be broadly categorized into two subsets: N1-state neutrophils (CD95^+^) that promote inflammation and tissue damage through the production of reactive oxygen species (ROS), damage-associated molecular patterns (DAMPs), neutrophil extracellular traps (NETs), and pro-inflammatory cytokines; and N2-state neutrophils (CD206^+^) that contribute to inflammation resolution and tissue repair (40, 41). We investigated the impact of GLSP on neutrophil polarization in CIA mice using flow cytometry (**Supplementary Figure S2A**). Compared to naive CIA mice, the vehicle group exhibited an increased proportion of N1-state neutrophils and a decreased proportion of N2-state neutrophils among total Ly-6G^+^ neutrophils population in the hind paw samples. GLSP treatment reversed this shift in Ly-6G^+^ neutrophil polarization (**Figures 4A–F**). Furthermore, N1-state neutrophil levels in the vehicle group negatively correlated with pain thresholds, a correlation not observed in the GLSP group (**Figures 4G–J**). Conversely, N2-state neutrophil levels positively correlated with pain thresholds in the GLSP group but not in the vehicle group (**Figures 4K–N**). These findings suggest that N1-state neutrophil accumulation within the synovium contributes to pain in RA, and that GLSP may alleviate this pain by promoting an N2 anti-inflammatory phenotype in synovial neutrophils.

### 3.4 GLSP extracts target “PTGS2,” “MMP9,” “XIAP,” and “MMP3” to exert therapeutic effects against RA

**Figure 5:**
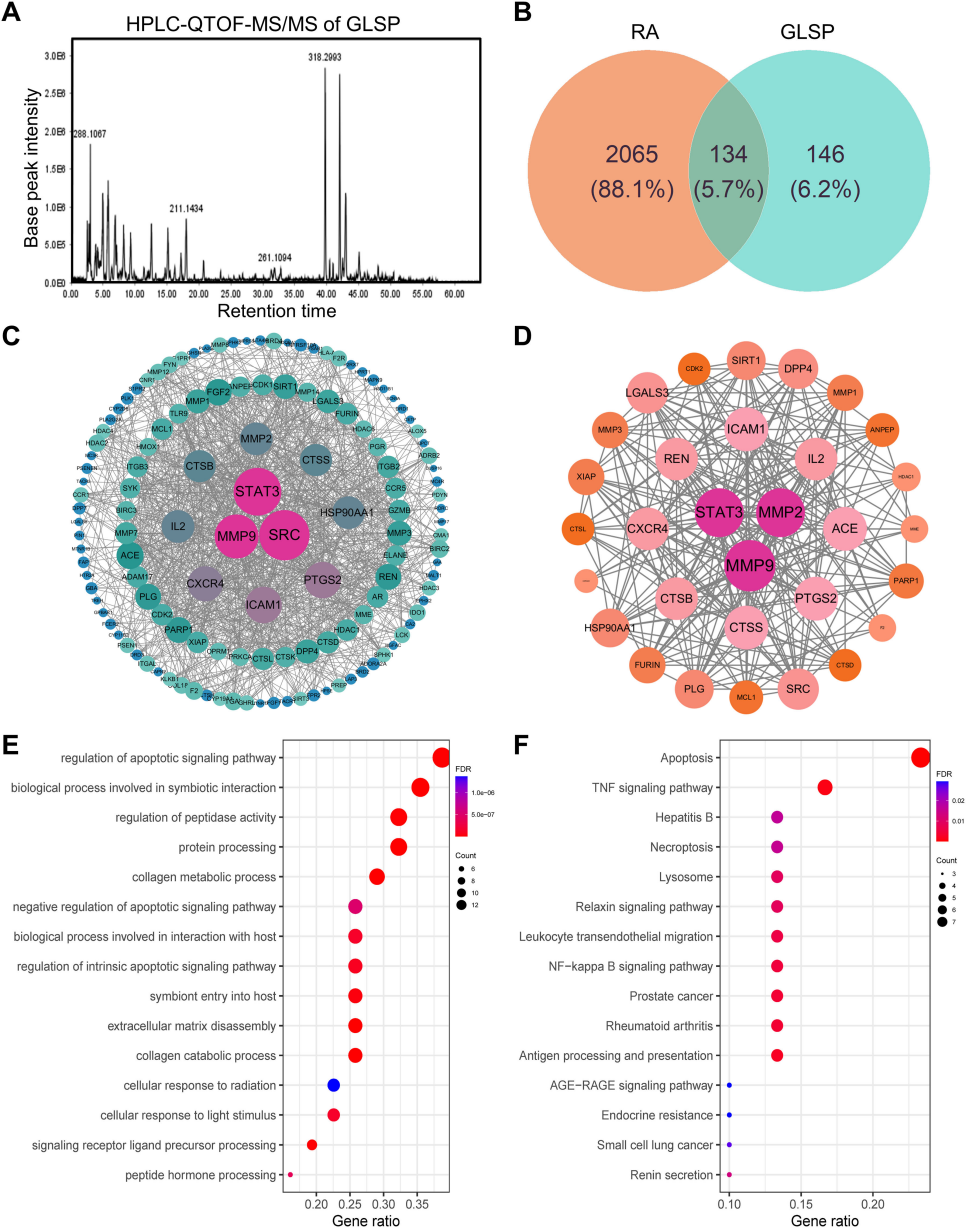
Identify the intersection targets shared by RA and GLSP for biological function analysis. **(A)** Total ion chromatogram of GLSP in positive ion mode. **(B)** 134 intersection targets of RA and GLSP. **(C)** The PPI network of intersection targets between disease targets of RA and the target proteins of active components in GLSP. **(D)** Key targets of GLSP for the treatment of RA were identified through CentiScaPe analysis utilizing Degree, Closeness, and Betweenness algorithms. **(E–F)** GO biological functional enrichment analysis and KEGG pathway analysis of key targets of GLSP in the treatment of RA.

**Figure 6:**
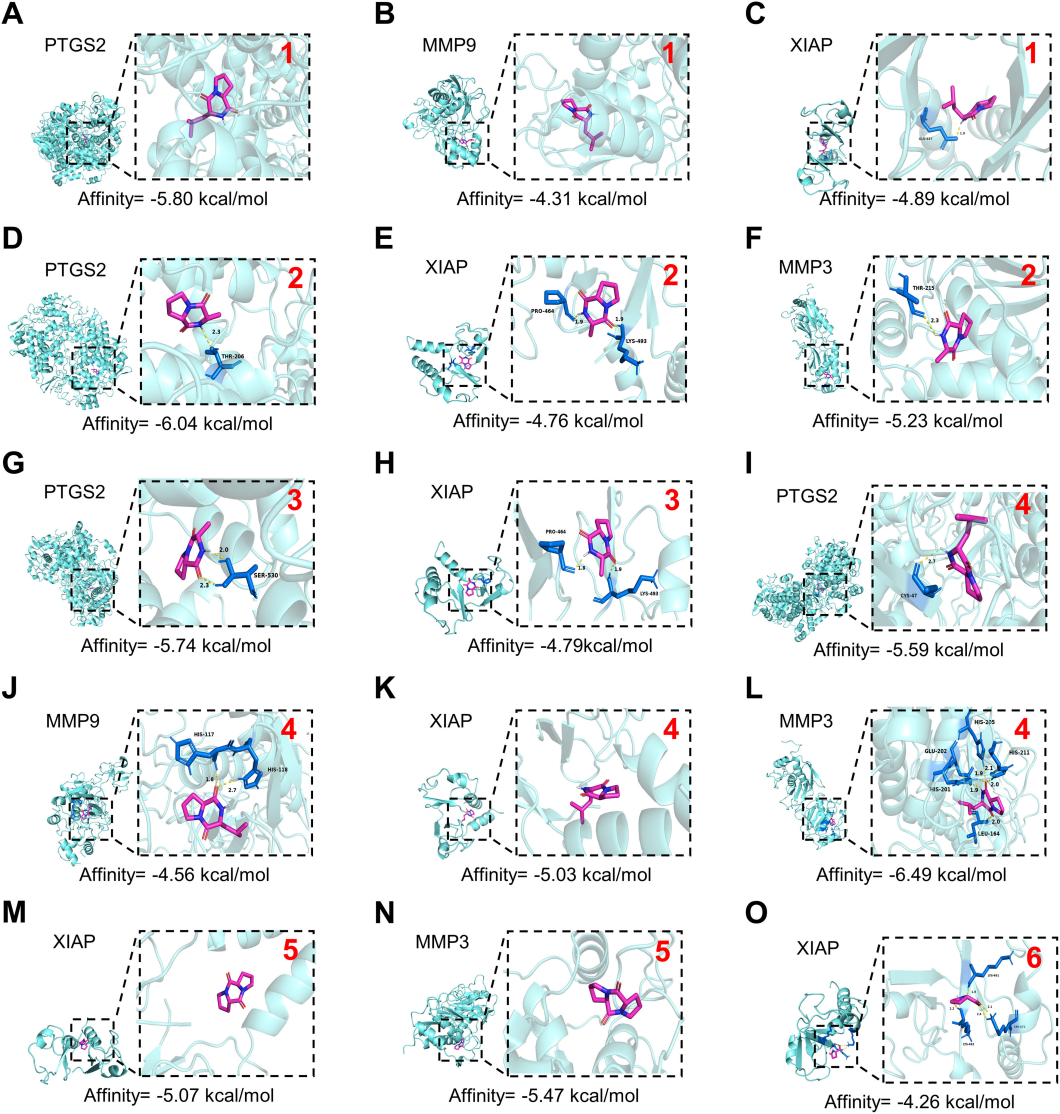
Docking patterns of core targets and active compounds of GLSP. **(A–O)**. Molecular docking analysis predicts the binding of compounds extracted from GLSP to key targets of RA. The red numerals in the upper right corner correspond to GLSP compounds 1–6 listed in **Table 1**. The crystal structure of PTGS2 (PDB ID: 3HS5), MMP9 (PDB ID: 1L6J), XIAP (PDB ID: 8W59) and MMP3 (PDB ID: 1G49) was obtained from the Protein Data Bank.

GLSP, as a commonly used health food, is primarily consumed daily in the form of tea or medicinal wine made by soaking it in water or alcohol. To investigate the active components and corresponding targets of GLSP for the treatment of RA, we extracted the components of GLSP using the aforementioned methods and conducted HPLC-QTOF-MS/MS analysis to identify the main components and their structures (**Figure 5A**, **Table 1**). Subsequently, we obtained 146 targets of GLSP extracts from Swiss Target Prediction database and Super-PRED database. RA-related targets were identified from GeneCards database and DisGeNET database. After removing overlapping targets from the databases, we selected targets with relevance scores greater than the median. Eventually, we identified 2065 high-confidence targets for RA. Based on the Venn diagram, we screened out 134 intersection targets and utilized Cytoscape software to construct the GLSP-RA target PPI network (**Figures 5B, C**). Following the construction of the GLSP-RA intersection target construction of the protein-protein interaction (PPI) network, we filtered the data based on CentiScaPe analysis, identifying 32 key targets according to Degree, Closeness, and Betweenness algorithms (**Figure 5D**). We then performed Gene Ontology (GO) and Kyoto Encyclopedia of Genes and Genomes (KEGG) analyses on these key targets, revealing their involvement in the top 15 biological processes and signaling pathways, among which the most significant is the TNF-α signaling pathway (**Figures 5E, F**). It is well established that the activation of the TNF-α signaling pathway plays a critical role in the progression of RA (42). The key targets linked to the TNF-α signaling pathway include *“PTGS2”*, *“MMP9”*, *“XIAP”*, *and “MMP3*”. Subsequently, we evaluated the binding affinity of the active components of GLSP extract for these targets through molecular docking simulations. The results demonstrate that stable interactions can be established between the active components and the targets. (**Figures 6A–O**). In summary, the active components of GLSP may achieve therapeutic effects through the interaction with these targets.

**Table 1:** Clarify the chemical constituents in GLSP.

No.	*t*_R_/min	Molecular formula	*m*/z (calculated)	*m*/*z* (measured)	Error (ppm)	Positive ion mode	Compound	Structure	Main fragment ion
1	17.20	C_11_H_19_N_2_O_2_	211.1447	211.1434	6.16	[M+H]^+^	gancidin W	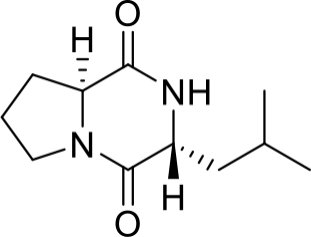	211.1430, 183.1486, 154.0732, 138.1272, 86.0960, 70.0645
2	6.92	C_8_H_13_N_2_O_2_	169.0977	169.0966	6.50	[M+H]^+^	3-methyl-octahydropyrrolo[1,2-a]pyrazine-1,4-dione	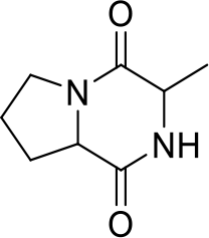	169.0965, 141.1022, 113.1077, 96.0807, 70.0644
3	12.59	C_10_H_16_N_2_O_2_	197.1290	197.1279	5.58	[M+H]^+^	3-(propan-2-yl)-octahydropyrrolo[1,2-a]pyrazine-1,4-dione	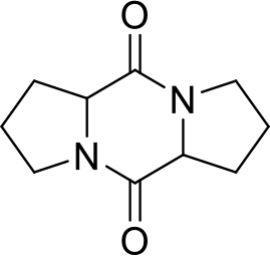	197.1276, 169.1330, 154.0734, 124.1117, 98.0597, 70.0645
4	5.74	C_9_H_14_N_2_O_3_	199.1083	199.1070	6.53	[M+H]^+^	Cyclo(Pro-Thr)	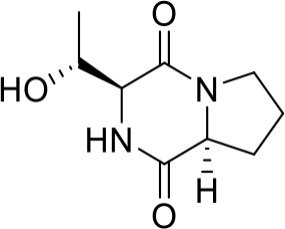	199.1077, 181.0967, 153.1015, 125.0708, 70.0644, 56.0492
5	9.30	C_10_H_14_N_2_O_2_	195.1134	195.1122	6.15	[M+H]^+^	1,7-diazatricyclo[7.3.0.03,7]dodecane-2,8-dione	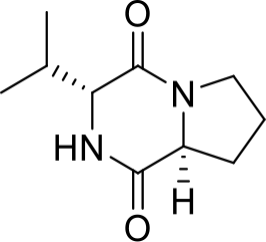	195.1115, 165.1015, 151.1225, 123.0911, 98.0592, 70.0643
6	4.15	C_5_H_8_NO_3_	130.0504	130.0493	8.45	[M+H]^+^	pidolic acid	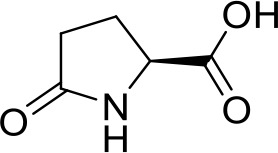	130.0490, 102.0556, 84.0437, 56.0490

The components of GLSP extract were identified using HPLC-QTOF-MS/MS.

### 3.5 GLSP extract reverses the pro-nociceptive properties of TNF-α- activated neutrophils

**Figure 7:**
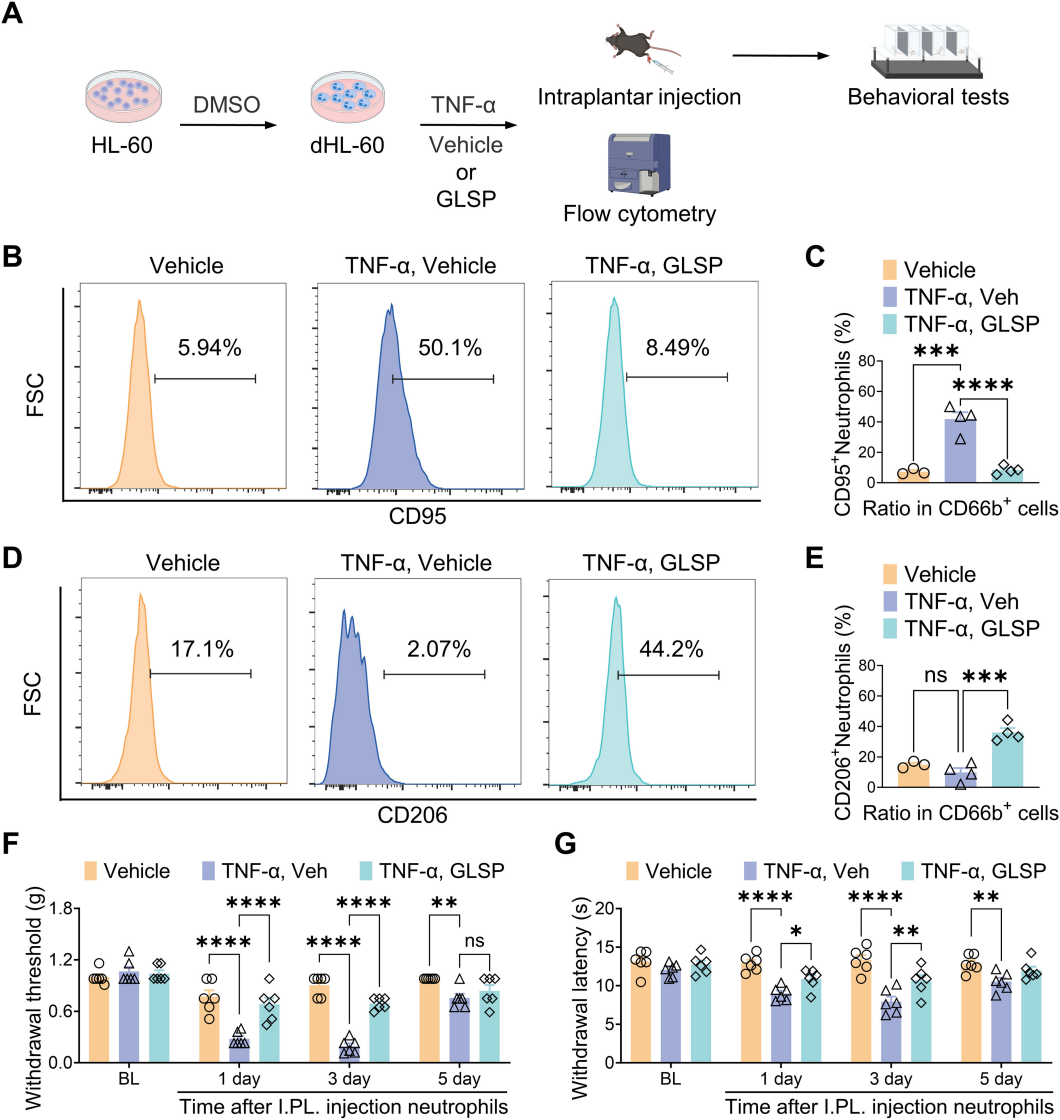
GLSP attenuates TNF-α-induced nociceptive effects in neutrophils by reversing their N1 polarization. **(A)** Schematic representation of the experimental protocol designed to investigate the nociceptive effects of neutrophil N1 and N2 polarization states. **(B, C)** TNF-α challenge increased the proportion of CD95^+^ neutrophils in dHL-60 cells, an effect reversed by Co-treatment with GLSP extract, as shown by flow cytometry. **(D, E)** Co-treatment with GLSP extract reversed the TNF-α-challenged reduction in the proportion of CD206^+^ neutrophils in dHL-60 cells. **(F, G)** Intraplantar injection of TNF-α-challenged neutrophils induced mechanical allodynia and thermal hyperalgesia. Co-treatment with GLSP extract attenuated this effect. Data are mean ± SEM. *p < 0.05, **p < 0.01, ***p < 0.001, and ****p < 0.0001, one-way ANOVA assay followed by Tukey’s *post hoc* test **(C, E)**, two-way ANOVA assay followed by Tukey’s *post hoc* test **(F, G)**. NS, no significance.

The *in vivo* experimental results indicate that GLSP primarily exerts its therapeutic effects by reducing the pro-inflammatory polarization of neutrophils. Network pharmacology analyses demonstrate that GLSP mainly influences the TNF-α signaling pathway. It is well established that the pro-inflammatory cytokine TNF-α is highly expressed in RA, which triggers immune dysregulation and contributes to the onset of RA (42). Research suggests that the immune cascade mediated by TNF-α-activated neutrophils is significant in the inflammation seen in RA (43). However, the mechanisms through which neutrophils promote RA-related pain have not been clearly defined. To further investigate the mechanisms by which neutrophils contribute to RA-related pain, we utilized the human neutrophil cell line HL-60. HL-60 cells were differentiated into neutrophil-like cells (dHL-60) through dimethyl sulfoxide (DMSO) induction, acquiring the functional characteristics of neutrophils (44). Subsequently, we treated dHL-60 cells with TNF-α to simulate an *in vitro* RA environment (**Figures 7A**, **Supplementary Figure S3A**). Flow cytometric and western blot analysis revealed that TNF-α stimulation significantly increased the proportion of CD95^+^ (N1) subpopulation among CD66b^+^ neutrophils, while simultaneously decreasing that of the CD206^+^ (N2) subpopulation when compared to the control group. Co-culturing TNF-α-treatment neutrophils with GLSP extract reversed this trend (**Figures 7B–E**, **Supplementary Figure S2B**). This finding corroborates our *in vivo* experimental conclusion that, under RA conditions, neutrophils preferentially polarize towards the N1 state, whereas GLSP treatment promotes polarization towards the N2 state. Furthermore, we performed an adoptive transfer of these differently treated dHL-60 cells into the plantar region of naïve recipient mice (**Figure 7A**). Behavioral tests demonstrated that TNF-α-treated neutrophils induced mechanical allodynia and thermal hyperalgesia in the recipient mice compared to the vehicle control group. Notably, co-treatment with GLSP extract alleviated the pain-inducing effects of these TNF-α-treated neutrophils (**Figures 7F, G**). In summary, these experimental results further confirm that TNF-α-activated neutrophils exhibit a polarized N1 state with nociceptive effects, while GLSP extract co-treatment can mitigate these pain-inducing effects.

### 3.6 GLSP extract reverses the pain-inducing effects of TNF-α- activated neutrophils by inhibiting levels of ROS

**Figure 8:**
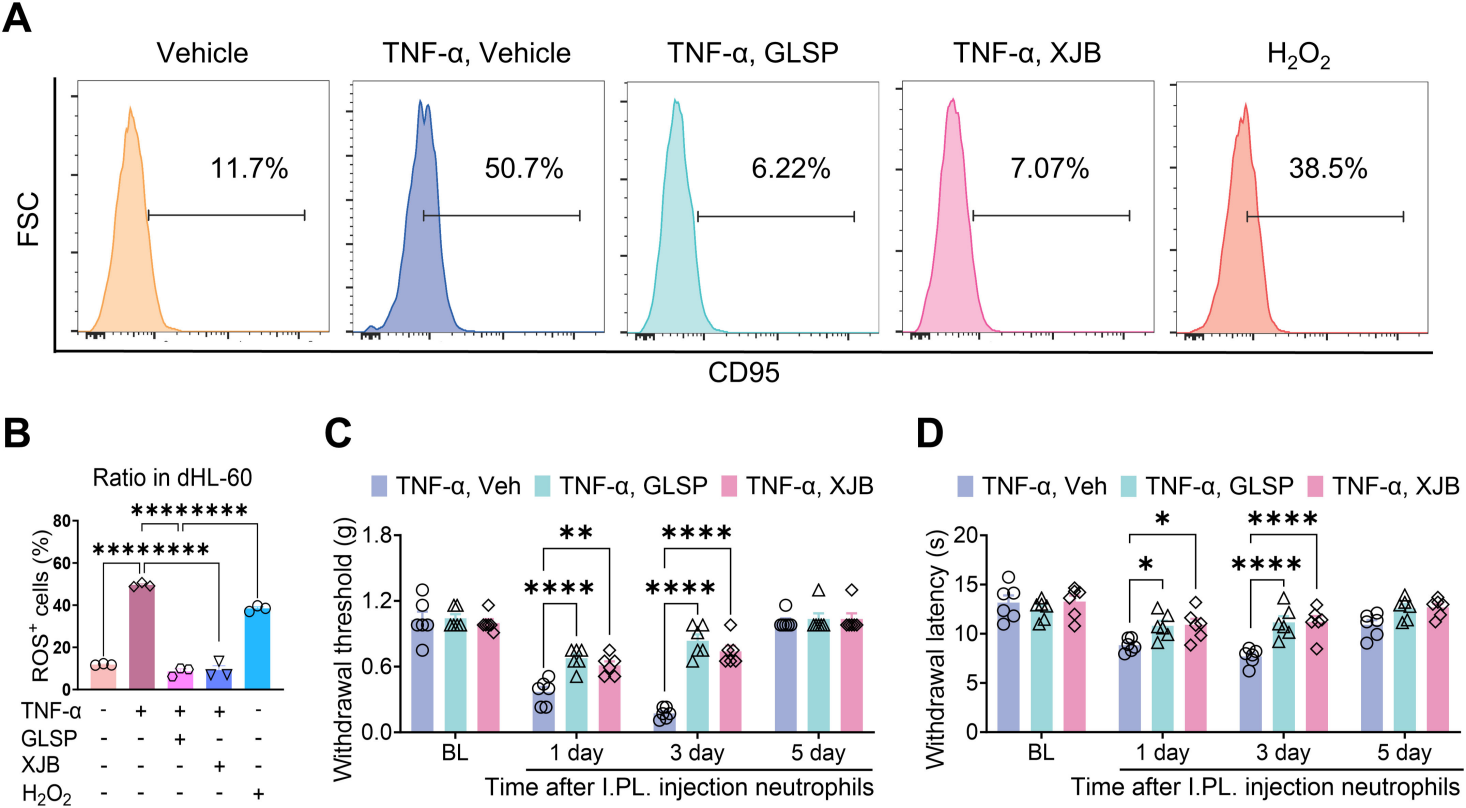
GLSP attenuates TNF-α-induced nociceptive effects in neutrophils by inhibiting the production of ROS. **(A)** The gating strategy employed to ROS levels in TNF-α-treatment neutrophils, along with representative plots of ROS levels across different groups, is presented. **(B)** TNF-α or H_2_O_2_treatment increases the proportion of ROS^+^ neutrophils in dHL-60 cells *in vitro*, while co-treatment with GLSP or XJB-5–131 extract reverses this increase. **(C, D)** The intraplantar injection of TNF-α-treatment neutrophils induces mechanical allodynia and thermal hyperalgesia. Co-treatment with either GLSP extract or XJB-5–131 mitigates this effect. Data are mean ± SEM. *p < 0.05, **p < 0.01, ***p < 0.001, and ****p < 0.0001, one-way ANOVA assay followed by Tukey’s *post hoc* test **(B)**, two-way ANOVA assay followed by Tukey’s *post hoc* test **(C, D)**.

TNF-α signaling can induce oxidative stress by increasing reactive oxygen species (ROS) levels, activating the caspase cascade, and altering mitochondrial function (45). Prior research indicates that under pathological conditions, abnormally elevated levels of ROS in neutrophils contribute to the progression of RA by activating pro-inflammatory signaling pathways, promoting the release of neutrophil extracellular traps (NETs), and initiating immune cell cascades (46, 47). Furthermore, increased ROS production within neutrophils at inflammatory sites can activate adjacent sensory neurons and enhance the activity of sensory neuron channel proteins, thereby inducing pain sensitization (48). The GLSP extract affects several targets within the TNF-α signaling pathway, including “*PTGS2*”, “*MMP9*”, “*XIAP*” and “*MMP3*” (**Figures 5F**, **6A–O**). Previous research indicates that these genes primarily regulate the production and release of ROS (49–51). Consequently, we propose that GLSP exerts its therapeutic effects by intervening in the TNF-α signaling pathway to reduce ROS levels. To validate this mechanism, we measured ROS levels in neutrophils treated with various conditions using flow cytometry, using H2O2 and the ROS-scavenging agent XJB-5–131 as controls. *In vitro* experimental data indicated that compared to the control group, TNF-α stimulation significantly increased the proportion of ROS^+^ dHL-60 cells, while co-treatment with GLSP inhibited ROS production (**Figures 8A, B**, **Supplementary Figure S3B**). GLSP demonstrated a ROS-clearing effect similar to that of XJB-5-131 (**Figures 8A, B**). Furthermore, we transferred these differently treated dHL-60 cells via intraplantar administration to naive recipient mice. Behavioral tests showed that both GLSP extract and XJB-5–131 co-treatment alleviated the pain-inducing effects of TNF-α-treated neutrophils (**Figures 8C, D**). In summary, we propose that N1-state neutrophils primarily exert pain-inducing effects through elevated ROS levels, while GLSP primarily functions as an analgesic agent by reducing these ROS levels.

### 3.7 GLSP alleviates RA-related pain by reducing levels of ROS in the paws of CIA mice

**Figure 9:**
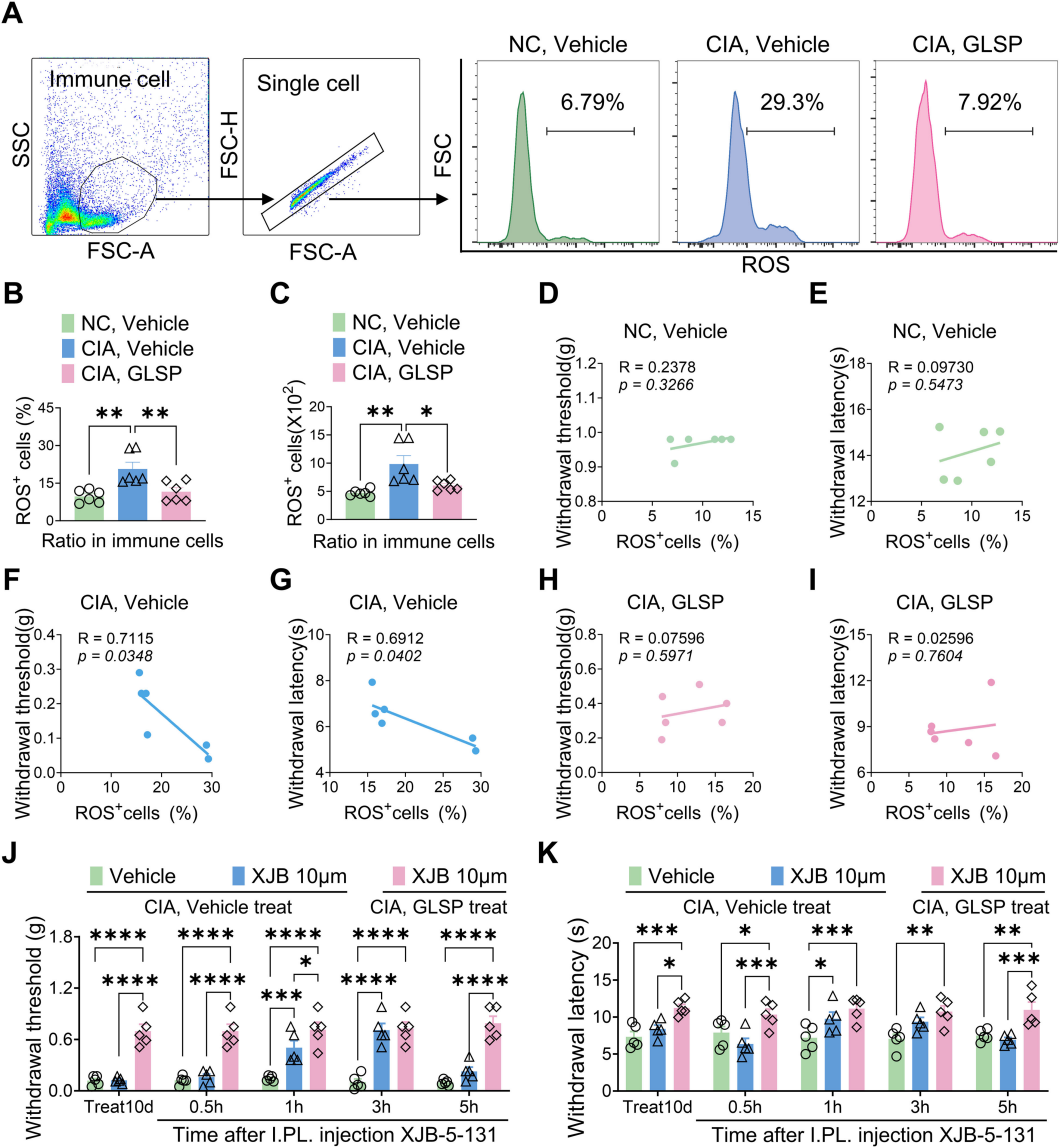
GLSP exerts therapeutic effects by reducing ROS levels in the paws cell of mice with CIA. **(A)** The gating strategy employed to ROS levels in paw immune cells, along with representative plots of ROS levels across different groups, is presented. **(B, C)** *In vivo* studies demonstrate that GLSP treatment reduces both the proportion and number of ROS-positive cells within the affected paw tissues of CIA mice. **(D–I)** The ratio of ROS-positive cells in the vehicle group, but not in the GLSP or naive control group, negatively correlates with mechanical and thermal pain thresholds. **(J, K)** The ROS scavenger XJB-5–131 alleviates mechanical allodynia and thermal hyperalgesia in CIA mice treated with the vehicle, but does not exert a similar effect in mice treated with GLSP. Data are mean ± SEM. *p < 0.05, **p < 0.01, ***p < 0.001, and ****p < 0.0001, one-way ANOVA assay followed by Tukey’s *post hoc* test **(B, C)**, Pearson correlation analysis **(D–I)**, two-way ANOVA assay followed by Tukey’s *post hoc* test **(J, K)**.

To further validate these findings, we measured ROS generation in hind paw samples from mice across different treatment groups (**Figure 9A**). The results revealed that type II collagen immunization significantly enhanced ROS generation, as evidenced by an increased proportion of ROS^+^ cells in the hind paw samples. Notably, GLSP treatment markedly reversed the elevated ROS levels in CIA mice (**Figures 9B, C**). Furthermore, the proportion of ROS-positive cells in the paws of vehicle-treated CIA mice positively correlated with mechanical allodynia and thermal hyperalgesia, a trend not observed in naive mice or GLSP-treated CIA mice (**Figures 9D–I**). Additionally, intraplantar injection of the ROS-targeting scavenger XJB-5–131 on day 10 post-vehicle treatment alleviated mechanical allodynia and thermal hyperalgesia in CIA mice, confirming that the ROS pathway is a key factor in RA-related pain. Moreover, XJB-5–131 did not increase the pain threshold in GLSP-treated CIA mice (**Figures 9J, K**). In summary, both *in vitro* and *in vivo* experimental results demonstrate that GLSP exerts RA-specific analgesic effects by targeting the TNF-α pathway, thereby mitigating neutrophil oxidative stress.

## 4 Discussion

Previous studies have demonstrated that alterations in immune cell states and disturbances in immune responses play a critical role in the pathogenesis of joint inflammation and pain associated with rheumatoid arthritis (RA) (2). However, the specific molecular mechanisms involved remain inadequately understood. Our earlier research indicated that neuro-immune interactions are significant contributors to pathological pain (10–13, 44–47). This study reveals, for the first time, a novel mechanism involving neutrophils that is closely linked to RA-related joint inflammation and pain through pharmacological, behavioral, and biochemical approaches. Our findings suggest that *Ganoderma lucidum* spore powder (GLSP) can effectively alleviate the symptoms of RA-related pain by reversing the pro-inflammatory phenotype of neutrophils and inhibiting their mediated inflammatory responses.

Our research elucidates the critical role of neutrophils in the pathology of RA-related pain. Neutrophils are the earliest responders and most abundant immune cells in the joints affected by RA (15, 17, 18). Recent evidence indicates that alterations in the immune microenvironment during disease progression induce the polarization of neutrophils into N1 or N2 phenotypes, with corresponding pro-inflammatory or anti-inflammatory functions in various disease pathologies (16). Our previous studies have shown that neutrophils account for up to 80% of the synovial fluid cell in RA patients, predominantly in the N1 phenotype. Furthermore, the levels of N1-state neutrophils within the synovial fluid positively correlate with pain scores in RA patients (data not published). In this study, we observed significant accumulation of neutrophils in the paw tissues of collagen-induced arthritis (CIA) mice, accompanied by a marked increase in the N1-state neutrophils. Notably, the proportion of N1-state neutrophils in the paws of CIA mice positively correlates with the degree of mechanical allodynia and thermal hyperalgesia. Importantly, oral administration of GLSP promotes the transition of neutrophils in the paw tissues of CIA mice from an N1 polarized state to an N2 polarized state, thereby exerting anti-inflammatory and analgesic effects. This finding suggests for the first time that the accumulation of N1-state neutrophils in the local joint microenvironment may play a significant role in the pathogenesis of RA-related pain.

Our research elucidates the relationship between N1 pro-inflammatory polarization of neutrophils and the activation of the TNF-α signaling pathway in the context of RA. We extracted the active components of GLSP and characterized their specific structures using HPLC-QTOF-MS/MS. Using network pharmacology approaches, we analyzed the target sites of the primary active components of GLSP in the treatment of RA and performed KEGG and GO functional enrichment analyses. The results indicate that GLSP primarily targets the TNF-α signaling pathway. It is well established that the expression of the pro-inflammatory cytokine TNF-α is elevated in RA patients, and TNF-α-activated neutrophils play a crucial role in the immune cascade associated with this condition (42). Therefore, we incubated neutrophils with TNF-α *in vitro* to simulate RA conditions. The results demonstrated that TNF-α stimulation polarized neutrophils into the N1-state, leading to pain hypersensitivity following their transfer to recipient mice. However, co-incubation with GLSP extracts reversed TNF-α-induced N1 polarization in neutrophils and their nociceptive effects. In conclusion, our findings suggest that neutrophils may display pro-nociceptive characteristics due to TNF-α activation in the synovial fluid under RA conditions, while GLSP appears to reverse these nociceptive effects by regulating the TNF-α signaling pathway.

Our research demonstrates that neutrophils in the paws of CIA mice regulate RA-related pain through the production of ROS. Previous studies have indicated that the activation of the TNF-α signaling pathway induces intracellular ROS production (45). Under pathological conditions, the activation of neutrophils leads to the production and release of ROS (52). In affected joints, the generation and release of ROS can result in synovial damage, thereby triggering an inflammatory response (17, 18). In gouty arthritis, ROS generated by neutrophils in affected joints can directly activate TRPA1^+^ neurons, contributing to pain (48). As the most abundant leukocytes in synovial fluid, neutrophils have been shown to promote synovial inflammation in RA patients through ROS generation; however, the specific role of ROS in RA-related pain remains unclear (16). This study provides *in vitro* evidence that TNF-α stimulation significantly increases ROS levels in neutrophils. Reducing ROS levels in neutrophils using either GLSP extracts or the ROS scavenger XJB-5–131 can diminish their pro-nociceptive effects. Consistent with this, ROS levels in the paw tissues of CIA mice are significantly elevated compared to those in the sham control group and positively correlate with the severity of pain in the mice. Additionally, local injection of the ROS scavenger XJB-5–131 effectively alleviates hyperalgesia in the vehicle-treated CIA mice, while CIA mice treated with GLSP do not exhibit any additional pain relief. These results indicate that GLSP alleviates RA-related pain by decreasing ROS production by neutrophils. Future studies should further investigate how ROS promote the upregulation of TRPV1 channel proteins and increase the release of CGRP in DRG sensory neurons, thereby mediating hyperalgesia.

Our research elucidates the role and mechanisms of GLSP in the treatment of RA-related pain. *Ganoderma lucidum* and its extracts have been utilized in traditional Chinese medicine for thousands of years, primarily for their immune modulation and antioxidant properties (21). Recent studies have demonstrated that the active components of *Ganoderma lucidum* exhibit multiple therapeutic effects on RA, including reversing the dysregulation of synovial immune cells, inhibiting fibroblast proliferation and angiogenesis, and promoting osteoblast formation (53). However, it remains uncertain whether Ganoderma spores can alleviate RA-related pain. This study found that oral administration of GLSP significantly reversed inflammation and pain hypersensitivity in the paws of CIA mice. First, behavioral and immunohistochemical data indicated that GLSP treatment alleviated pathological damage, mechanical allodynia, and thermal hyperalgesia in the paws of CIA mice. Second, flow cytometry analysis revealed that GLSP treatment significantly reduced the accumulation of neutrophils in the joints of CIA mice. Third, GLSP reversed the pro-inflammatory (N1) polarization of neutrophils in the paw tissues of CIA mice. Fourth, GLSP and its extracts inhibited the generation of ROS in neutrophils stimulated by TNF-α *in vitro* and in the joints of CIA mice *in vivo*. In summary, GLSP demonstrated significant anti-inflammatory and analgesic effects in the CIA model, suggesting its potential value in the treatment of RA-related pain.

Our research highlights the clinical translational potential of GLSP in treating RA-related pain. Traditional disease-modifying antirheumatic drugs (DMARDs) are well-known for their adverse effects, which include cytopenia, transaminase elevation, poor tolerability (fatigue, nausea, and central nervous system side-effects), rash, and, rarely, interstitial lung disease or liver damage (54). Although biological agents have fewer of these side effects, they can still carry risks, such as increased susceptibility to infections and elevated cholesterol levels (54). Additionally, their high costs impose a significant economic burden on patients over the long term (54). Therefore, there is an urgent need to explore alternatives to traditional medications for treating RA. Dietary therapy, an essential component of traditional Chinese medicine, has been shown to be effective in the treatment and prevention of various diseases (19). Due to its recognized therapeutic properties, *Ganoderma lucidum* has emerged as one of the most extensively studied chemopreventive agents and functional foods, demonstrating high biological safety (22). This study found that oral administration of GLSP effectively alleviates RA-related inflammation and pain. Moreover, its water-soluble extracts contain sufficient active compounds to exert anti-inflammatory effects, indicating that it can be easily consumed, either taken directly or steeped in water. Being cost-effective, GLSP imposes a smaller burden on patients. These findings suggest that GLSP holds promise as a novel health food for the treatment and prevention of RA.

This study has several limitations (1). Our immunofluorescence data indicate that type II collagen immunization increases the expression of TRPV1 and CGRP, whereas GLSP treatment reverses this effect. These findings suggest that GLSP may regulate the excitability of DRG sensory neurons under RA conditions. However, it remains unclear whether GLSP can directly act on sensory neurons to exert regulatory effects. Future research investigating whether GLSP exerts long-term effects by altering the transcriptome of sensory neurons will be important. (2) Our study demonstrates that GLSP regulates the production of ROS in neutrophils by modulating the TNF-α signaling pathway. However, its specific functional patterns in this process remain unclear and warrant further investigation. (3) In addition to inhibiting ROS production, GLSP may regulate neutrophil activity in RA condition through additional mechanisms. Further studies are needed to explore the role of various neutrophil mechanisms in the pathology of RA-related pain and the potential therapeutic effects of GLSP on these mechanisms.

In summary, our research findings indicate that the accumulation and activation of neutrophils in joint drive RA-related pain, representing a crucial advancement in understanding the pathology of RA. Further studies demonstrate that neutrophils regulate RA-related pain through the production of ROS. Notably, GLSP, recognized as a health food, exerts analgesic effects on RA-related pain by inhibiting the N1 state polarization of neutrophils and reducing ROS generation. Consequently, this study offers mechanistic insights into the pathophysiological processes underlying RA-related pain and the potential development of new applications of GLSP for its treatment.

## Data Availability

The original contributions presented in the study are included in the article/**Supplementary Material**. Further inquiries can be directed to the corresponding authors.
