# Supplementary material for: Ganoderma lucidum spore powder alleviates rheumatoid arthritis-associated pain hypersensitivity through inhibiting accumulation, N1 polarization, and ROS production of neutrophils in mice
Source: Front Immunol. 2025 Apr 30;16:1569295. doi: 10.3389/fimmu.2025.1569295 (PMC12075414; doi:10.3389/fimmu.2025.1569295)
Supplement: Supplementary file 1 [file DataSheet1.docx]

Supplementary Material

**Supplementary Table 1. A list of reagents for this study**

| Reagent or resource | Source | Identifier |
| --- | --- | --- |
| Chemicals, peptides, and recombinant proteins | | |
| Human TNF-alpha Recombinant Protein | Thermofisher | 300-01A-1MG |
| H2DCFDA | MCE | HY-D0940 |
| XJB-5-131 | MCE | HY-129460 |
| Penicillin-Streptomycin Liquid | Solarbio | P1400 |
| RPMI Medium 1640 | Solarbio | 10491 |
| DMEM | Solarbio | 11995 |
| Red Blood Cell Lysis Buffer | Solarbio | R1010 |
| Software and algorithms | | |
| GraphPad Prism v9.5.1 | GraphPad Software | <https://www.graphpad.com/> |
| Flowjo v10.8.1 | Flowjo v10 | <https://www.flowjo.com/solutions/flowjo> |
| R (version 4.4.1) | R Development Core Team | <https://cran.r-project.org/> |
| ImageJ 1.53t | Wayne Rasband and contributors National Institutes of Health, USA | <http://imagej.nih.gov/ij> |
| Gradient 1.51.1.16 | BIOSEB | [http://www.bioseb.com](http://www.bioseb.com/) |
| PEAKS Studio 8.5 | Bioinformatics Solutions Inc | <https://www.bioinfor.com/peaks-85-release/> |
| NIS-Elements Viewer 5.21.00 | Nikon | <https://www.microscope.healthcare.nikon.com/> |
| Discovery Studio 2021 | BIOVIA | <https://www.3ds.com/> |
| Pyrx-0.8 | SOURCEFORGE | <https://pyrx.sourceforge.io/> |
| AutoDock Vina39 | Molecular Biology Building | <https://vina.scripps.edu/> |

**
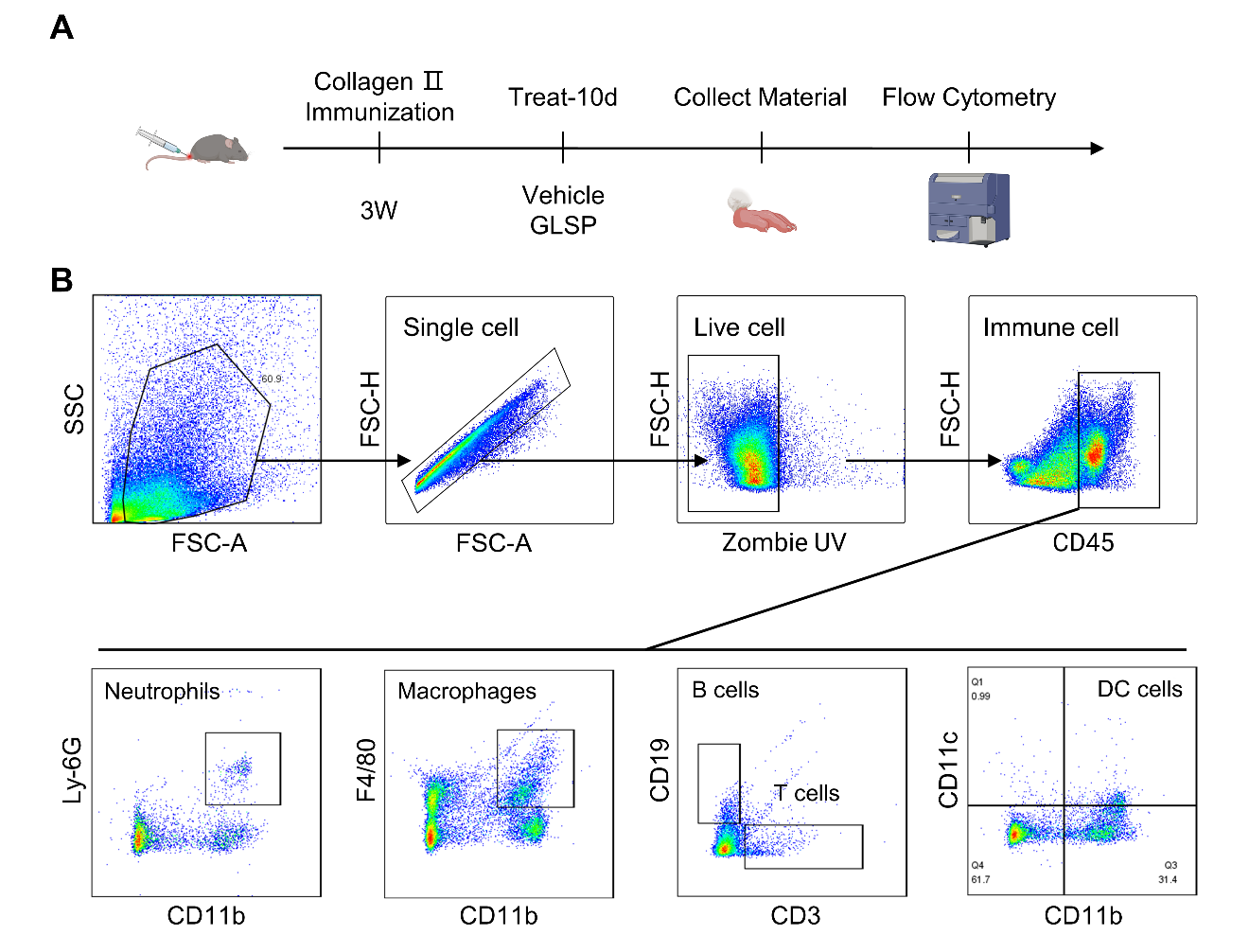
**

**Figure S1. Gating strategy for flow cytometry analysis for subtyping immune cells in mouse paw samples.**

(A) Processing protocol and sample collection time points for CIA mice. (B). Total cells extracted from mouse paw samples were initially gated on a forward scatter (FSC-A) / side scatter (SSC-A) plot, followed by the removal of cellular debris. Subsequently, specific markers were employed to analyze changes in viable neutrophils (CD45^+^CD11b^+^Ly-6G^+^), macrophages (CD45^+^CD11b^+^F4/80^+^), T cells (CD45^+^CD3^+^), B cells (CD45^+^CD19^+^), and dendritic cells (CD45^+^CD11b^+^CD11C^+^).

**
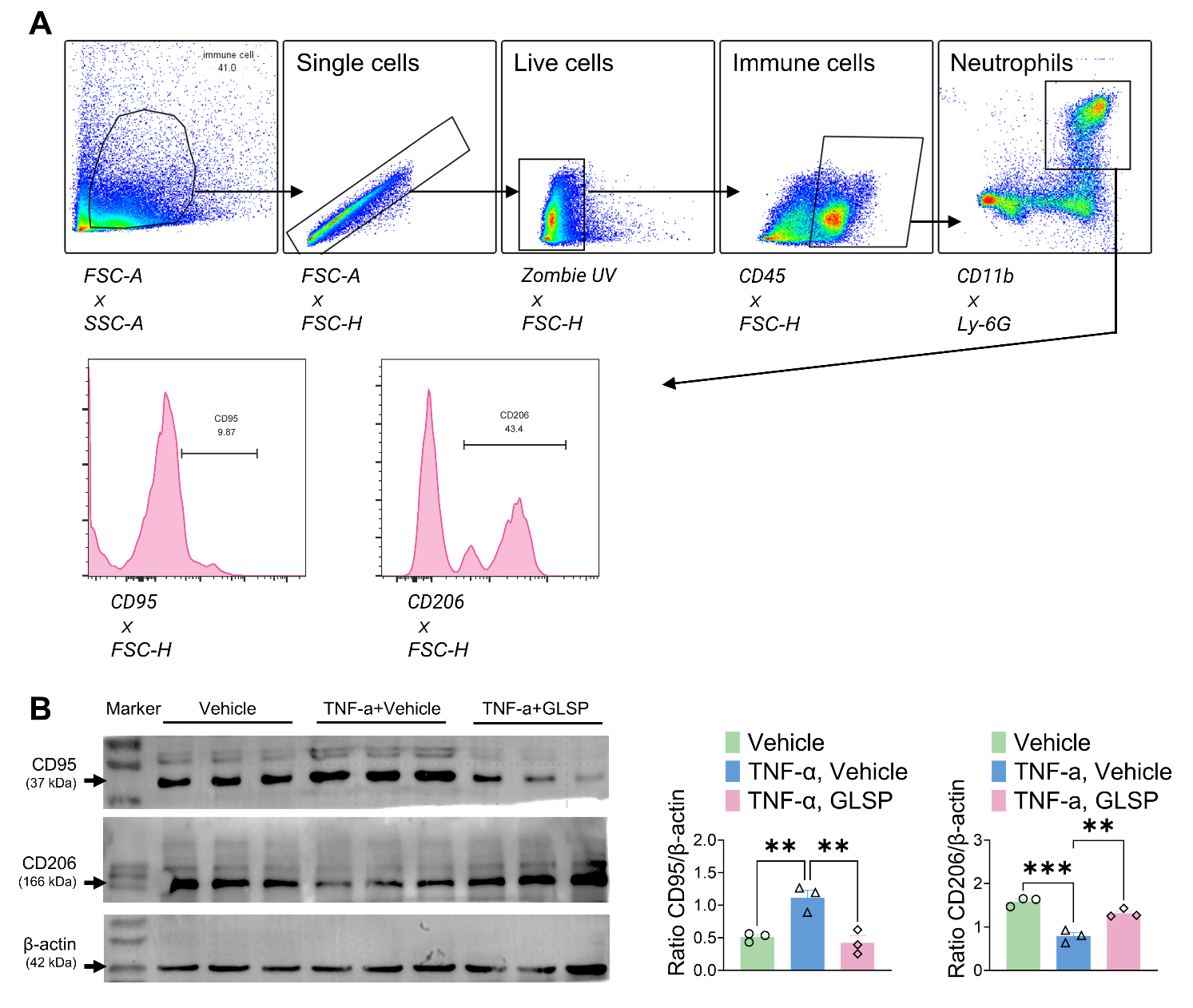
**

**Figure S2. Gating strategy for flow cytometry analysis for the polarization identification of neutrophils in mouse paw samples.**

(A) Total cells extracted from mouse paw samples were initially gated based on a forward scatter (FSC-A) and side scatter (SSC-A) plot, followed by the exclusion of cellular debris. Specific markers were then employed to analyze variations in the N1-state (CD45^+^CD11b^+^Ly-6G^+^CD95^+^) and N2-state (CD45^+^CD11b^+^Ly-6G^+^CD206^+^) subpopulations of viable neutrophils (CD45^+^CD11b^+^Ly-6G^+^). (B) Western blot analysis demonstrated that TNF-α treatment significantly increased CD95 expression while decreasing CD206 expression in dHL60 cells. These effects were reversed by GLSP co-treatment. Data are mean ± SEM. **p < 0.01, and ***p < 0.001, one-way ANOVA assay followed by Tukey's post hoc test (C, E).

**
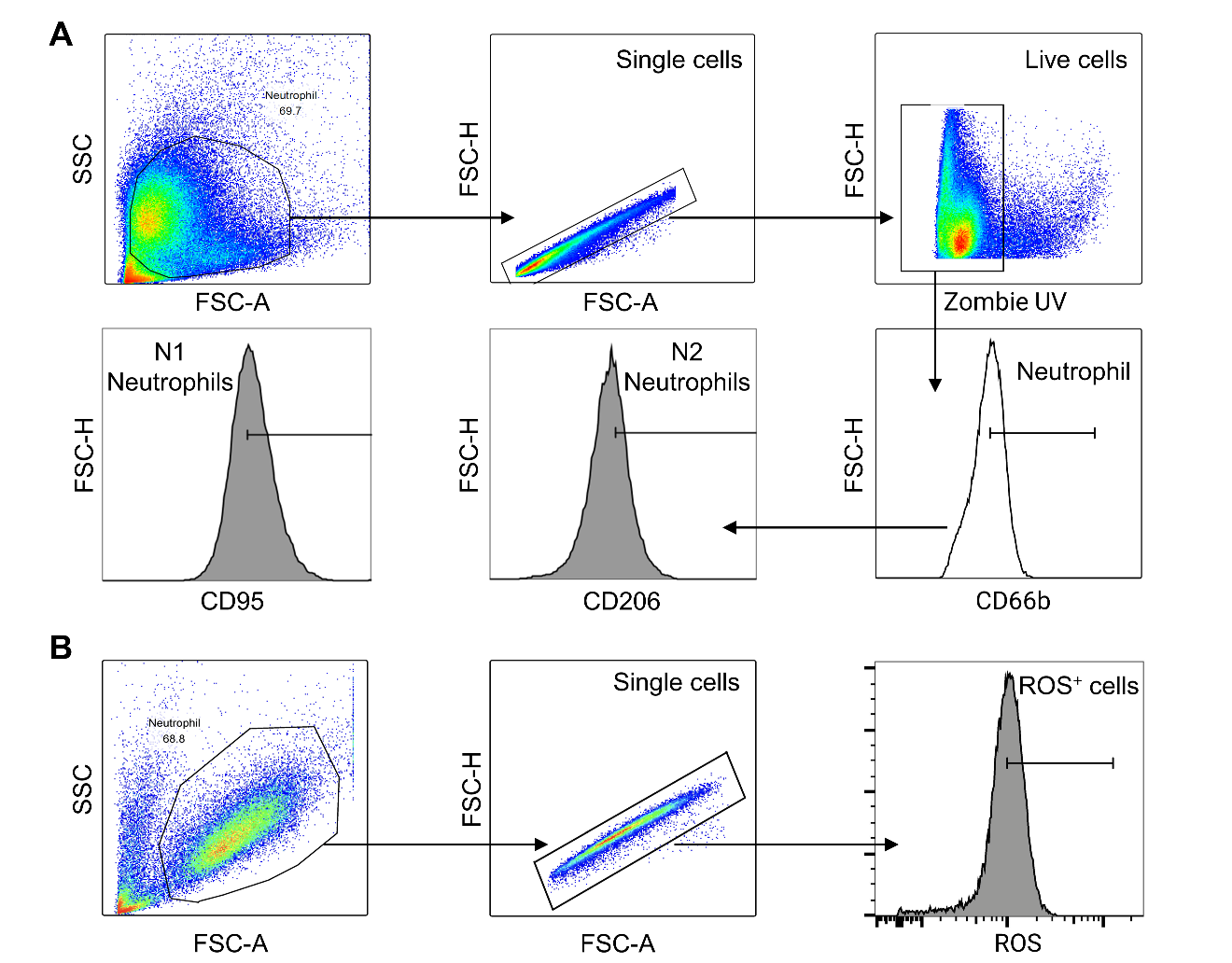
**

**Figure S3. The gating strategy used for flow cytometry analysis *in vitro* experiments with the dHL-60 cell.**

(A) TNF-α-treated dHL-60 cells were initially gated on a forward scatter (FSC-A) / side scatter (SSC-A) plot. This was followed by the removal of cell clumping and the delineation of the neutrophils (CD66b^+^) population. Subsequently, changes in the N1-state (CD66b^+^CD95^+^) and N2-state (CD66b^+^CD206^+^) subpopulations of neutrophils among different groups were analyzed. (B) TNF-α-treated dHL-60 cells were initially gated on a forward scatter (FSC-A) / side scatter (SSC-A) plot, followed by the removal of cell clumping. Subsequently, the levels of ROS in cells from different groups were analyzed.
